# A Rare Presentation of Tuberculosis: Multifocal Skeletal and Multisystemic Disease Evaluated with Imaging Findings

**DOI:** 10.5334/jbsr.2679

**Published:** 2022-04-07

**Authors:** Burak Günay, Büşra Özdemir, Fethi Emre Ustabaşioğlu

**Affiliations:** 1 Department of Radiology, Trakya University Faculty of Medicine, TR; 2 Department of Nuclear Medicine, Trakya University Faculty of Medicine, TR

## Abstract

**Teaching point:** Tuberculosis (TB) is a rare clinical disease in the musculoskeletal system. Multifocal bone involment and multisystemic spread of this disease is extremely uncommon and difficult to diagnose.

## Case History

**Figure 1:**
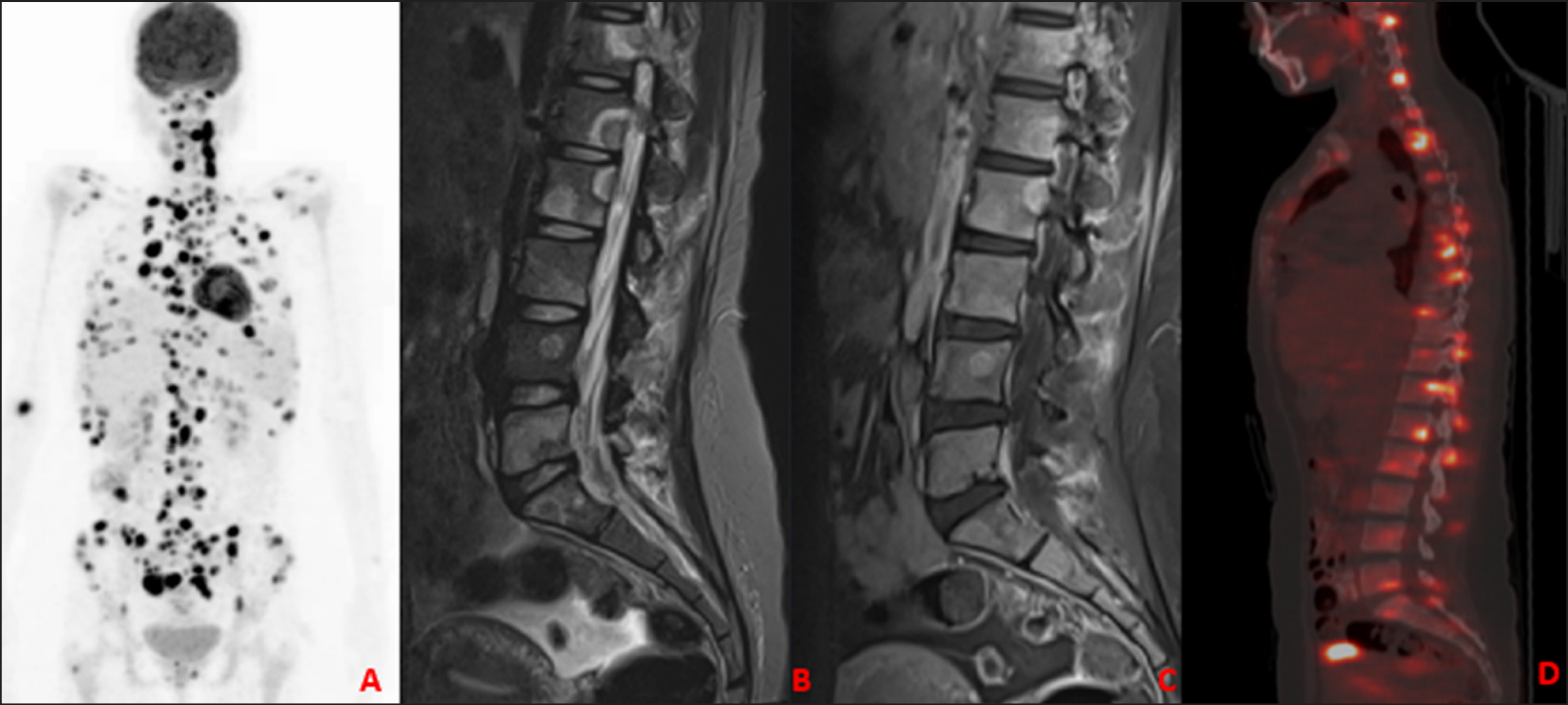


**Figure 2:**
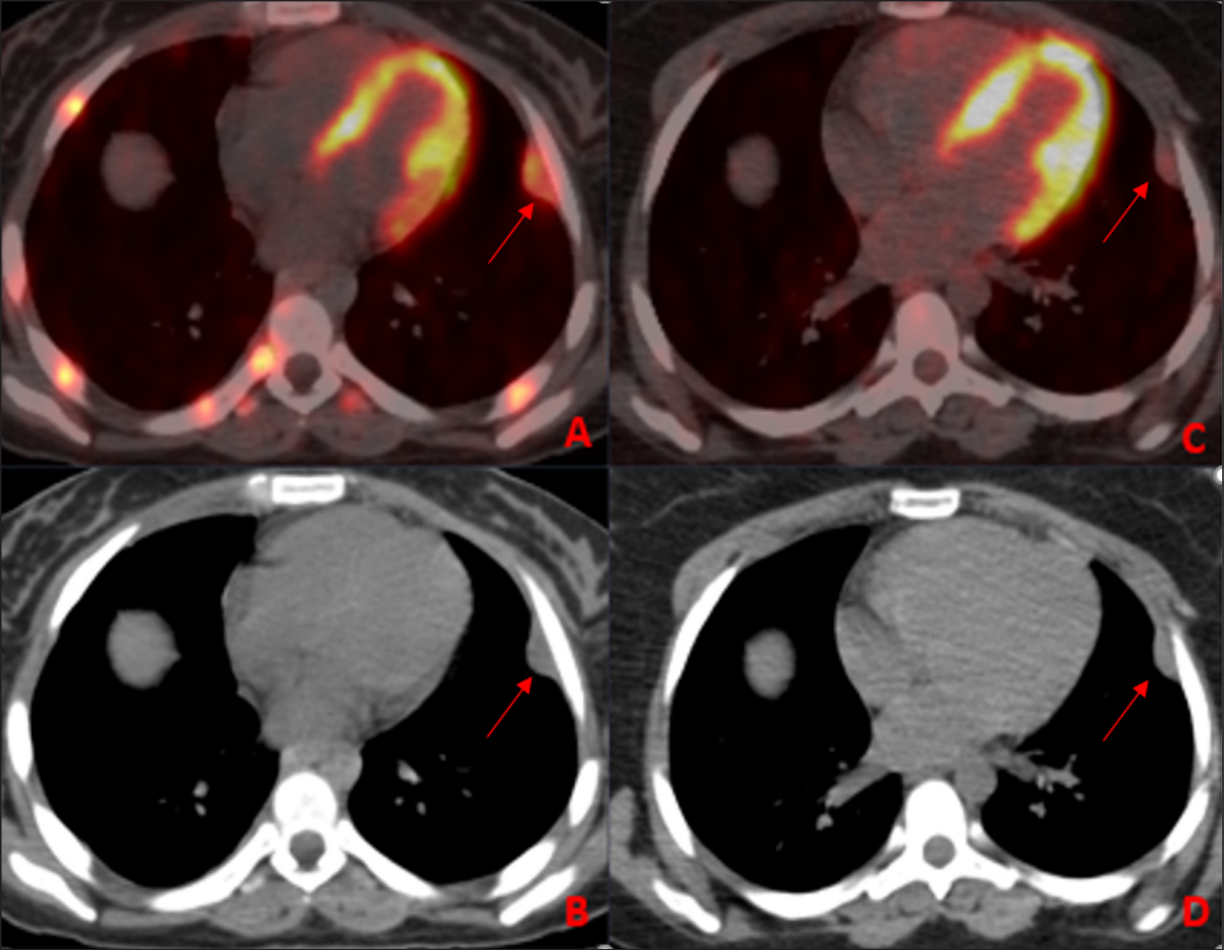


**Figure 3:**
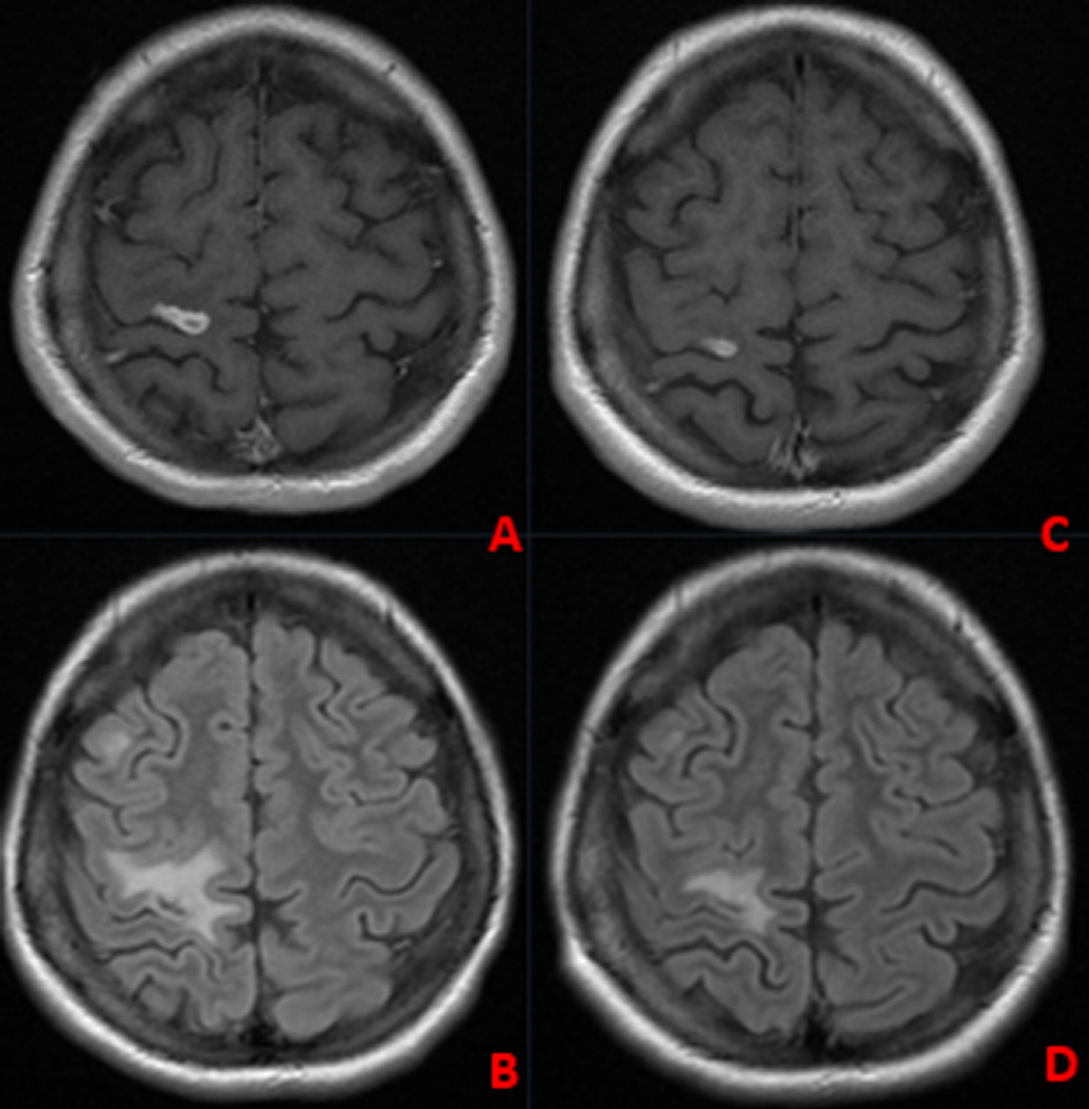


A 27-year-old female patient was admitted to Trakya University Hospital with a history of recurrent fever, weight loss, and intermittent lumbar back pain lasting for one month. The patient also reported persistent headache, chills, and night sweats. She has no history of TB exposure or chronic lung disease. Cranial and lumbar magnetic resonance imaging (MRI), thoracic and lumbar computed tomography (CT), and whole-body 18F-FDG PET/CT were taken upon her complaints (***Figures 1***, ***2***, ***3***).

Based on the symptoms and the examinations, meta-static disease or bone tuberculosis was suspected. Subsequently, to make the definitive diagnosis, open biopsy was performed under the L2 lamina and L2 vertebral bodies. Histopathological examination showed local sequestrum formation. Langerhans cells were detected with giant cells and dead bones, which are the characteristic features of TB disease.

## Comment

In this case report, which showed unusual radiological findings that were more indicative of malignancy or metastatic disease, a rare case of atypically disseminated multifocal skeletal and multisystemic TB was identified. Thus, due to the complex and atypic clinical findings of multifocal skeletal TB, it is easy to misdiagnose the disease [1].
